# Critical limb ischemia in a patient with systemic lupus erythematosus: a case report

**DOI:** 10.1186/s13256-019-2024-9

**Published:** 2019-04-25

**Authors:** Vito Damay, Wendy Wiharja, Raymond Pranata, Melisa Aziz

**Affiliations:** 1 Department of Cardiovascular, Siloam Hospital Lippo Village, Karawaci, Tangerang, Indonesia; 2 Faculty of Medicine, Universitas Pelita Harapan, Karawaci, Tangerang, Indonesia

**Keywords:** Critical limb ischemia, Systemic lupus erythematosus, Immune complex, Peripheral angioplasty

## Abstract

**Background:**

Peripheral vascular disease is the rarest vascular complication in systemic lupus erythematosus. Some theories propose that immune complexes may promote inflammation in the vessel, and disrupt it in a manner that may cause ischemia.

**Case presentation:**

A 14-year-old Asian girl presented with intermittent claudication as the chief complaint followed by discoloration of her left big toe for 2 weeks prior to admission. Her medical history showed that 1 month prior to admission she had photosensitivity, rash, and arthralgia, with positive antinuclear antibody test, positive anti-double-stranded DNA test, positive anti-ribosomal protein P, and complement C4 (7.4 mg/dL); she was diagnosed as having systemic lupus erythematosus and started therapy. A local examination of her left toe showed black discoloration, low pulsation, localized tenderness, and decreased sensation. Laboratory results showed C-reactive protein of 1.16 mg/dL and D-dimer of 2.28 uG/mL. A computed tomography angiogram showed near total occlusion of her popliteal artery; critical limb ischemia was confirmed. Peripheral arteriography was performed with invasive strategy. After the procedure, the flow was improved to distal, but there was inflammation in the vessel, so we decided to stop the procedure because of the risk of dissection. Our patient was given atorvastatin and warfarin, and we maximized her systemic lupus erythematosus therapy with prednisone. There were two follow-ups. The first follow-up was 1 week after the procedure. Our patient attended her first follow-up at our out-patient department with no symptoms and improvement in her toe’s discoloration; warfarin was stopped, and clopidogrel and cilostazol were added for thrombus prevention therapy, she was then scheduled for debridement. The second follow-up was done 2 months after the first follow-up and discoloration was improved. The third follow-up, 5 months after the second follow-up, showed improvement.

**Conclusion:**

Critical limb ischemia is a rare complication of systemic lupus erythematosus that requires immediate treatment. Due to our limited resources, we improvised a strategy to achieve the best possible outcome in our patient by using a combination of invasive treatment and medication.

## Background

Critical limb ischemia (CLI) is a disease caused by a sudden decrease in perfusion of the limbs which promotes clinical symptoms such as claudication and discoloration [1]. On the other hand, systemic lupus erythematosus (SLE) is an auto-immune disease which causes the body’s immune system to mistakenly attack its healthy tissues/organs [1]. The Swiss SLE Cohort Study (SSCS) showed that only 13.3% patients with SLE have vascular events as a complication [1], among them were: coronary heart disease (CHD) 8.3%, cerebrovascular disease (CVD) 5%, and peripheral arterial disease (PAD) 1.2% [2]. Many factors associate vascular events and SLE, including disease duration and metabolic comorbidities. The pathophysiology is still unclear [1, 2]. Immune complexes may play a significant role in the pathomechanism; a deposit of immune complex may promote an inflammatory response and lead to vasculitis, and this turn of events may reduce the flow in the vessel and then produce ischemia [2]. Several predictors such as high laboratory result for C-reactive protein (CRP), longer duration of disease, older age, history of known cardiovascular disease, and history of tobacco smoking may predict the incidence of peripheral vascular events in SLE [3].

This case report is about a 14-year-old Asian girl who was diagnosed as having SLE and treated for SLE, she then presented to our emergency department with symptoms of CLI. With the limited resources of our center, our management strategy was to improvise to achieve the best possible result. We review and elaborate our treatment plan in this case report; we hope that our experience will assist a center in a similar situation to treat a patient with the same condition.

## Case presentation

**Fig. 1:**
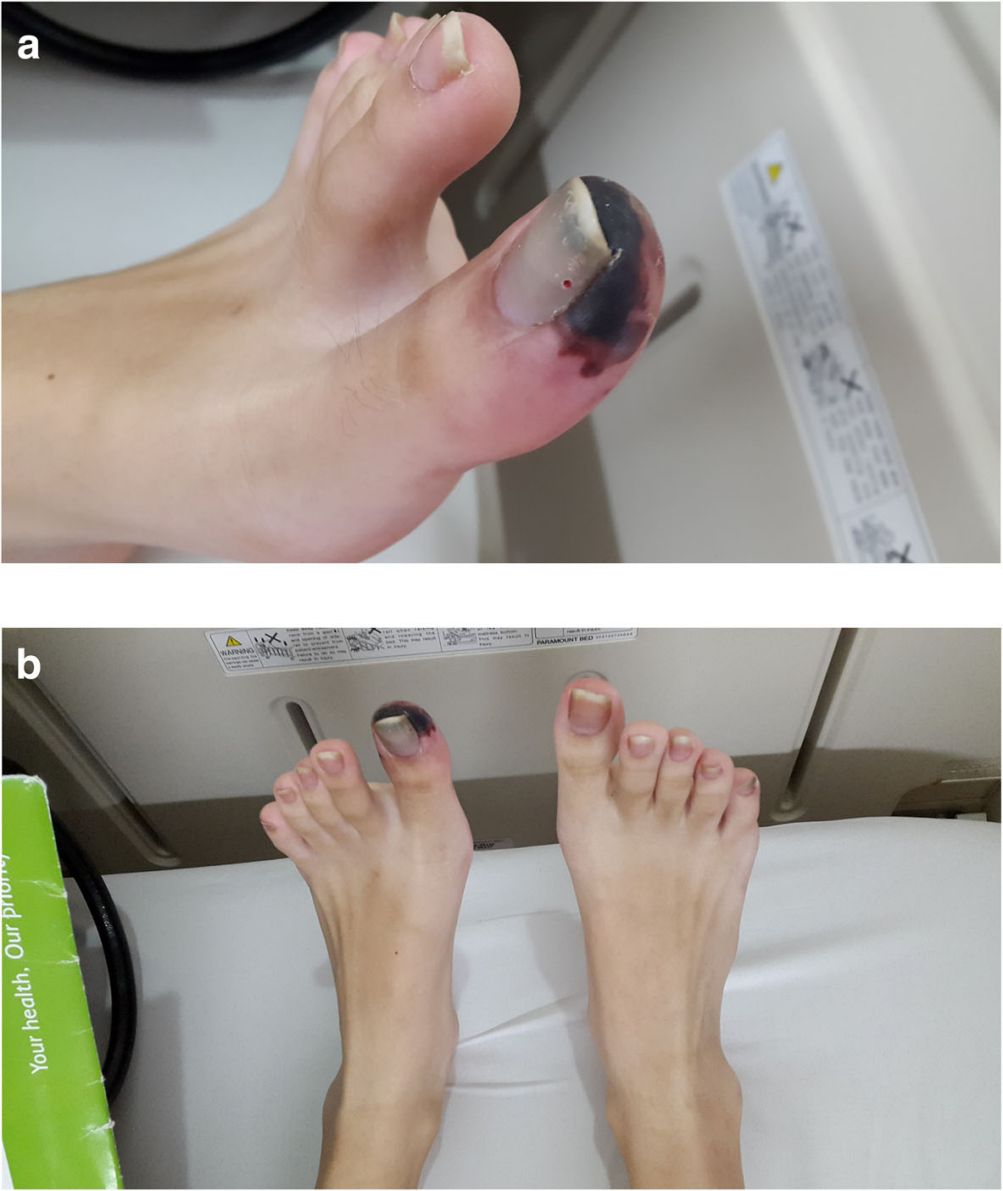
**a** Black discoloration, low pulsation, positive localized tenderness, and decreased sensation. **b** Both feet are shown for comparison

**Fig. 2:**
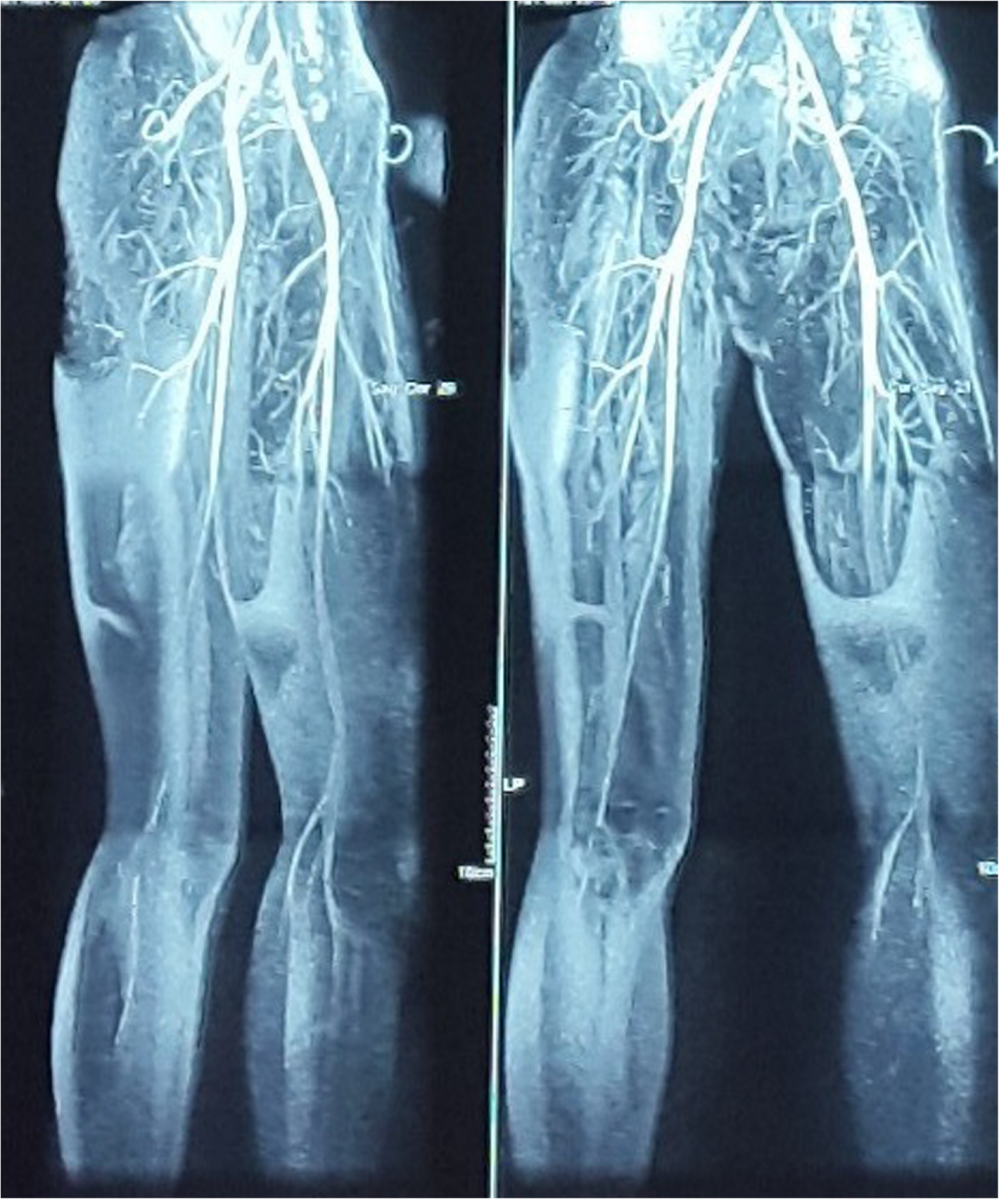
Patient’s computed tomography-angiography. Patient’s computed tomography-angiography showing near total occlusion of the popliteal artery

A 14-year-old Asian girl presented to our emergency department with intermittent claudication as a chief complaint and with discoloration of her left big toe of 2 weeks’ duration. The claudication was located around her left foot, worsening day by day, and it made her unable to walk properly and limited her physical activity. A physical examination was performed at our emergency department: her blood pressure was 110/70 mmHg, pulse was 80 beats per minute (bpm), respiratory rate was 20 times/minute, and her temperature was 37.3 °C. A localized examination was performed on the big toe of her left foot; it showed black discoloration, low pulsation, and positive localized tenderness (Fig. 1a, b). A neurological examination showed decreased sensation in the big toe of her left foot; motor function and physiologic reflexes were within normal limits and no pathological reflexes were found. Other physical examinations were unremarkable. Laboratory results showed CRP of 1.16 mg/dL and D-dimer of 2.28 uG/mL. We performed a computed tomography (CT) angiogram, and its result showed near total occlusion of the popliteal artery; CLI was confirmed (Fig. 2).

One month before admission, she had symptoms of photosensitivity, myalgia, arthralgia, and a rash around her face and she was hospitalized. Laboratory tests showed positive antinuclear antibody (ANA) test, positive anti-double-stranded DNA (DS-DNA) test, positive anti-ribosomal protein P (RIB), and complement C4 (7.4 mg/dL); she was diagnosed as having SLE and started on prednisone 5 mg twice a day as the main treatment. Social, environmental, and familial history were unremarkable. She did not smoke tobacco or consume alcohol. She had received no past relevant intervention.

**Fig. 3:**
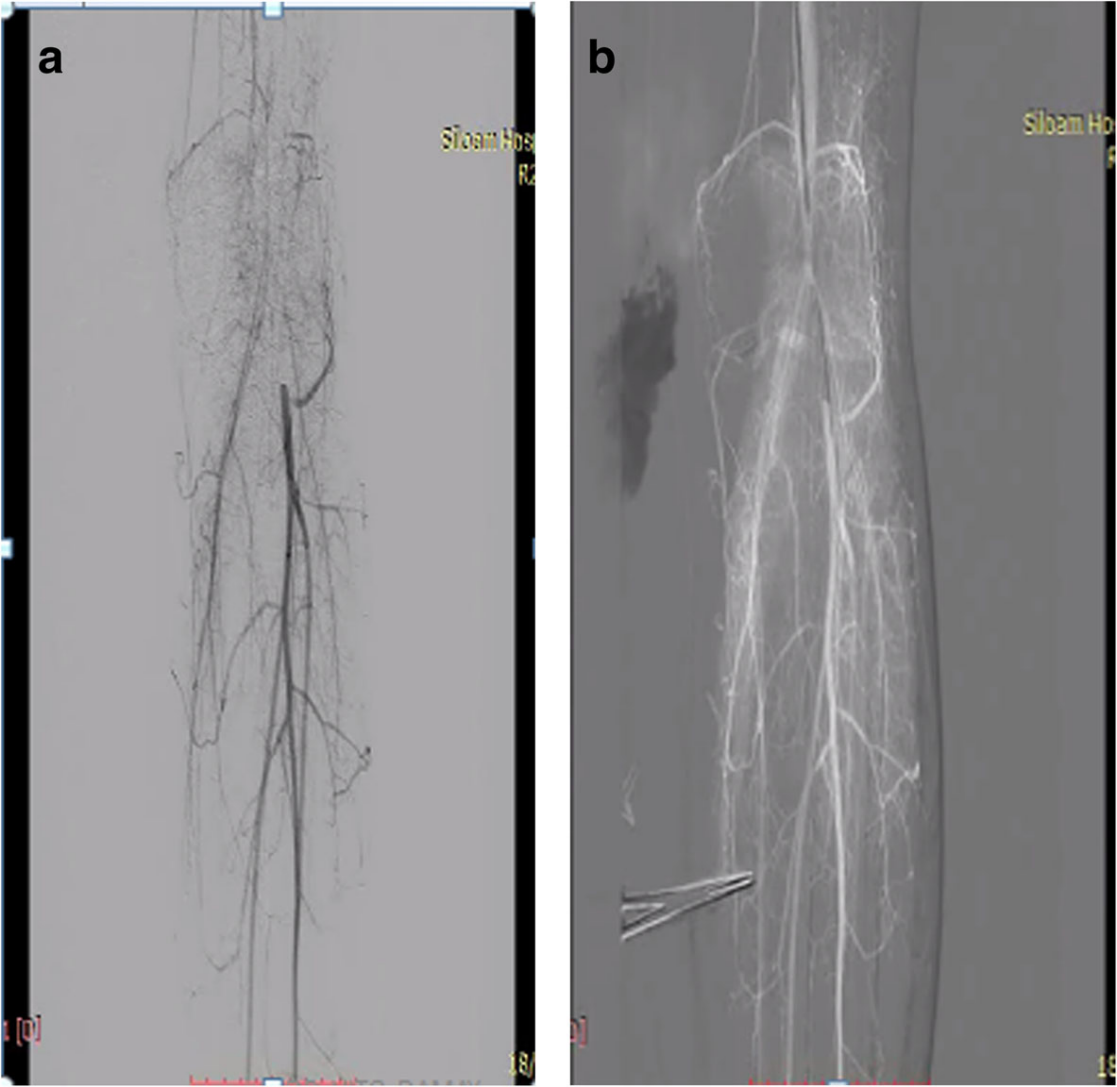
**a** Patient’s digital subtraction angiography and angiography pre-intervention. **b** Soft wire through the lesion

**Fig. 4:**
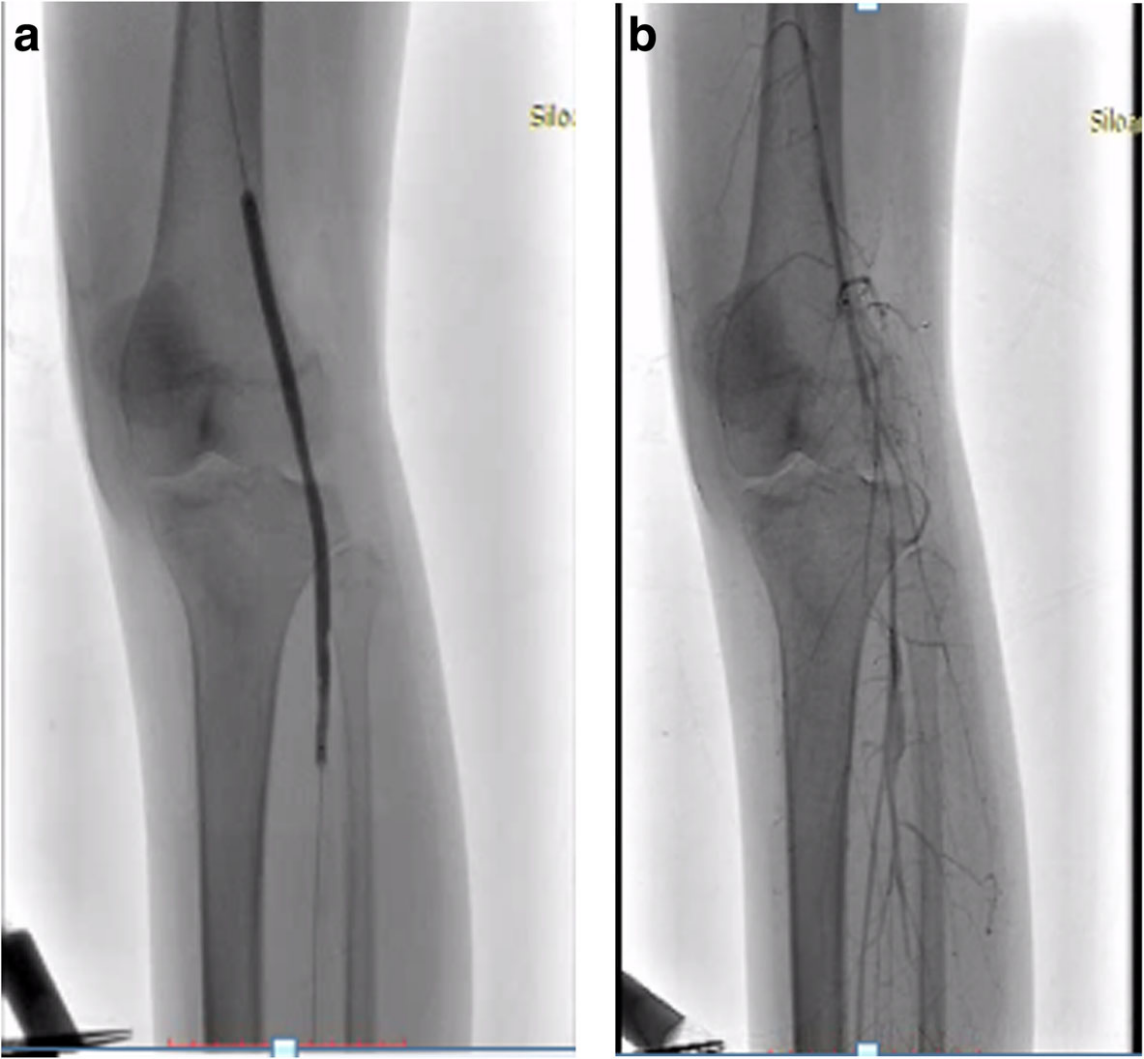
**a** Multiple plain old balloon angioplasty dilatations. **b** Flow after multiple plain old balloon angioplasty dilatation procedures

**Fig. 5:**
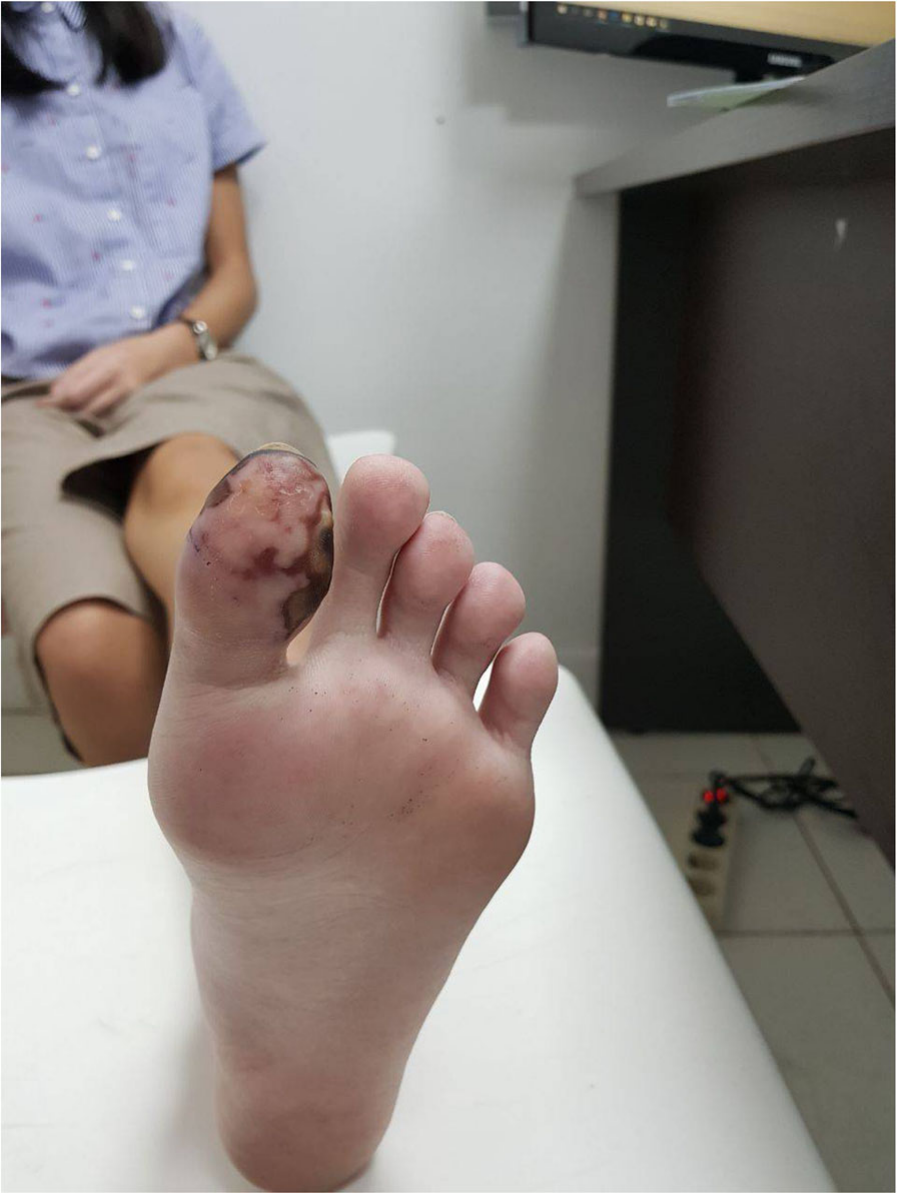
Patient’s left big toe on first follow-up. The first follow-up was 1 week after the procedure. The follow-up took place at our out-patient department; our patient’s left big toe showed improvement in discoloration and no symptoms of claudication

**Fig. 6:**
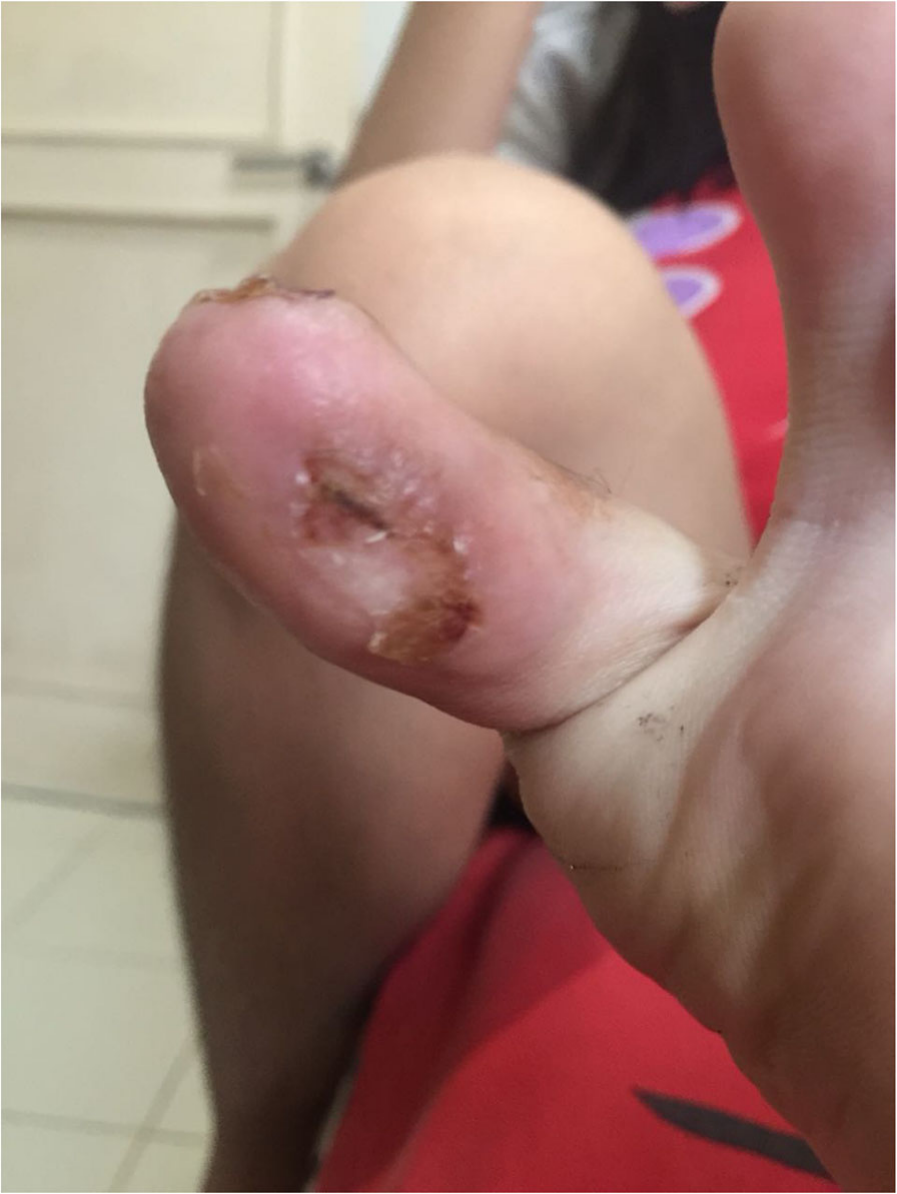
Patient’s left big toe on second follow-up. The second follow-up was 2 months after the first follow-up; the improvement in discoloration was greater than the improvement on the first follow-up

**Fig. 7:**
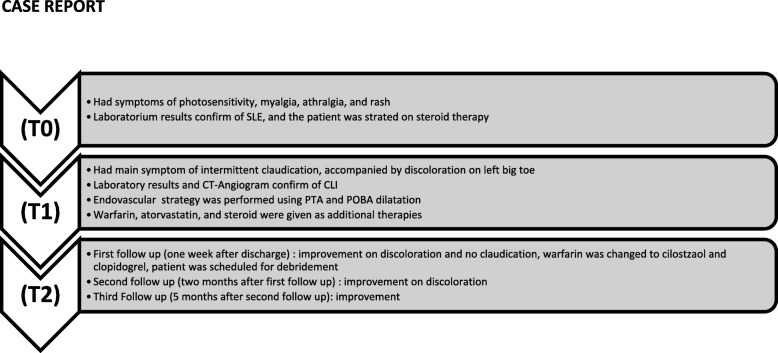
Timeline of the disease. *CLI* critical limb ischemia, *CT* computed tomography, *POBA* plain old balloon angioplasty, *PTA* percutaneous transluminal angioplasty, *SLE* systemic lupus erythematosus, *T0* 1 month prior to admission, *T1* on admission, *T2* on follow-up

Peripheral arteriography was performed with a goal to improve the flow; a soft wire smoothly went through the lesion (Fig. 3a, b). After multiple dilatations with an over-the-wire balloon, there was persistent recoil and significant stenosis although the flow was improved; however, the procedure was stopped since there was an inflammation of the vessel, which gave rise to a risk of dissection (Fig. 4a, b). Warfarin 10 mg, atorvastatin 40 mg, and prednisone 5 mg twice a day were given after the procedure, and she was discharged. She attended follow-up at our out-patient department (OPD) 1 week later, there was a little improvement in the discoloration, and she did not complain about claudication anymore (Fig. 5). We changed the warfarin to cilostazol 100 mg twice a day and clopidogrel 80 mg. She was also scheduled for debridement and told to come back 2 months later for a second follow-up. On the second follow-up, the improvement in discoloration was better than the improvement in the first follow-up (Fig. 6). A third follow-up, 5 months after the second follow-up, showed improvement in symptoms and we planned to do an angiography to make sure about the lesion (see timeline, Fig. 7).

## Discussion

This case report was about a 14-year-old Asian girl who was diagnosed as having SLE and was treated for SLE, she then presented to our emergency department with symptoms of CLI. A CT scan showed near total occlusion of her popliteal artery. We decided to apply invasive management with an endovascular approach combined with medications: warfarin, prednisone, atorvastatin, cilostazol, and clopidogrel. Follow-ups of our patient showed improvement both in discoloration and symptoms. Many studies in the literature, ranging from systematic review to meta-analysis, just described the association between SLE and peripheral vascular disease (PVD). In our case, with all the limitations we had, we managed to improvise and provide the best possible strategic management for our patient; we will elaborate the judgment and reasoning for our choice of treatment, which we hope may contribute to the medical literature because there are very few case reports. Also, by reading our case report, physicians who work in resource-limited centers like ours might gain a common understanding of improvised therapeutic strategy which will be of use when encountering a patient with the same condition [4].

PVD was clinically defined as one or more of the following: intermittent claudication, absent/unequal pulses, gangrene, or ischemic ulcers; PVD was sub-clinically defined as asymptomatic patients with Doppler abnormalities with > or = 50% reduction in diameter considered hemodynamically significant [2, 5]. SLE in this patient was diagnosed using American Rheumatology Association criteria for SLE; our patient had 4 out of 11 criteria and they were photosensitivity, positive ANA test, positive anti-DS-DNA test, and malar rash [2, 6]. CLI was diagnosed clinically by the presence of intermittent claudication and discoloration with an onset of 2 weeks, and from an angiography study which showed a near total occlusion of her popliteal artery [6, 7].

The inflammatory response which is caused by SLE played a significant role in producing the vascular event in this patient; on the other hand, PVD can also develop in conditions caused by accompanying comorbidity (steroid-related). The pathogenetic outcome of augmented atherosclerosis and proinflammatory environment may promote the initial step of this problem. Inside the vessel wall, *in situ* formation of vascular disease might be promoted by inflammatory products which are deposited as immune complexes. There are several auto-antibodies, produced by those immune complexes, which compromise the endothelial cells on the vessel as cytotoxic effector, this step has been implicated in the pathogenesis of several connective tissue diseases, predominantly vasculitis. Anti-endothelial cell antibodies (AECAs) and antineutrophil cytoplasmic autoantibodies (ANCAs) are well-known autoantibodies that are found in SLE. More than 80% of patients with SLE are positive for AECAs and the other 15–20% are positive for ANCAs. Interaction between immune complexes (AECAs or ANCAs) with Endothelial lining cells may attracts monocyte adhesion and induces secretion of chemoattractant proteins and cytokines, thus triggering vasculitis. Fibrinoid degeneration, intimal thickening, thrombosis, and sclerosis were identified and recorded in a histologic study. The principal manifestations of the disease were found to be associated with smaller-sized arteries manifested on peripheral vasculature [2, 6, 8].

The important point of our case is the choice of the treatment plan because many variables should be considered to gain the optimal result. In terms of CLI, limb saving, as the goal, can be achieved by either endovascular therapy (EVT) or bypass graft method depending on the main lesion. A multidisciplinary approach was taken with discussion among a vascular surgeon, cardiovascular department, and internal medicine department to decide on the best treatment plan for our patient. This case is the first in our center, with limited resources (patient’s economic problem and staffing in our center) and after discussion, the vascular team and the internal medicine department agreed that EVT using percutaneous transluminal angioplasty (PTA) might be the best treatment plan. PTA was chosen based on many considerations, they were: younger age, many collateral vessels, and esthetical reasoning. Multiple dilations with plain old balloon angioplasty (POBA) were needed because of significant recoil of the vessel; a POBA which is used in such conditions should be smaller in size. Even though the flow was improved, we stopped the procedure because the already inflamed vessel may have caused dissection. From the literature, we found that the location of the lesion and procedural timing are the major factors for deciding between EVT and bypass surgery. For the aortoiliac and femoropopliteal arterial areas, the therapeutic results of lower limb EVT including long-term outcomes have improved and become equivalent to those of bypass surgery. Ensuring a long-term blood supply and less necrotic area are important in terms of limb salvage, and for area below the ankle or the knee, bypass surgery has been considered as the golden standard. However, CLI is commonly associated with other general vascular conditions such as coronary arterial and cerebrovascular problems. The Bypass versus Angioplasty in Severe Ischaemia of the Leg (BASIL) study showed a comparable short-term result between EVT and bypass surgery. In emergency cases, EVT is more favorable to be applied than bypass surgery since this procedure is simpler and can be done under local anesthesia [7–9].

After we stopped the procedure, we decided to maximize the steroid and we also added an anti-coagulant and statins as the maintenance therapy. At 1-week follow-up after the procedure, our patient showed no symptoms and an improvement in discoloration, so we decided to add anti-platelets to prevent a possible further thrombus event. On the second follow-up (2 months after the procedure), discoloration was improved. We maximized the prednisone dose because almost all parts of the vessel and its collaterals were in an already inflamed condition; hoping an inprovement on the next angiography follow-up, the inflammation process will be reduced and we can do EVT procedure if only the symptoms are not improving in the future. Warfarin as the anti-coagulant, and cilostazol and clopidogrel as the anti-platelets were chosen as additional therapy after we did the procedure, since the endothelial lining of an already inflamed vessel is very thin and destroyed by immune complexes. Thinning of the endothelial layer may promote micro-bleeding and if this is conjoined with a hypercoagulable state promoted by inflammatory response, then a thrombus may be produced in the future; by using an anti-coagulant and anti-platelets we hoped this condition could be prevented. Atorvastatin as a high index statin was added to the maintenance therapy of anti-coagulant and anti-platelets; this high index statin promotes an anti-inflammatory effect which may reduce immune complex deposition. A debridement procedure has to be done to reduce the necrotic lesion which hinders the healing process [5, 10, 11].

## Conclusion

CLI as a manifestation of PVD is a rare complication in SLE. With limited resources, this condition is hazardous and needs immediate treatment. A multidisciplinary approach is required to achieve an optimal outcome. An invasive strategy as well as medications, such as anti-platelets, anti-coagulant, statin, and steroid are needed and should be given simultaneously to achieve a better outcome.

## Patient’s perspective

The patient felt horrible about her disease and did not know about the disease until we described it to her.

## Abbreviations

AECAs: Anti-endothelial cell antibodies
ANA: Antinuclear antibody
ANCAs: Antineutrophil cytoplasmic autoantibodies
BASIL: Bypass versus Angioplasty in Severe Ischaemia of the Leg
bpm: Beats per minute
CHD: Coronary heart disease
CLI: Critical limb ischemia
CRP: C-reactive protein
CT: Computed tomography
CVD: Cerebrovascular disease
DS-DNA: Double-stranded DNA
EVT: Endovascular therapy
OPD: Out-patient department
PAD: Peripheral arterial disease
POBA: Plain old balloon angioplasty
PTA: Percutaneous transluminal angioplasty
PVD: Peripheral vascular disease
RIB: Ribosomal protein P
SLE: Systemic lupus erythematosus
SSCS: Swiss SLE Cohort Study
